# Impact of *Artemisia selengensis* Turcz. Leaf Extract on Beer Brewing: Fermentation Dynamics, Flavor Compounds and Hypolipidemic/Antihyperuricemic Effects

**DOI:** 10.3390/molecules30193936

**Published:** 2025-10-01

**Authors:** Zeyu Li, Jiazhi Zhou, Chaoqun Ye, Jian Yang, Changli Zeng

**Affiliations:** Hubei Engineering Research Center for Protection and Utilization of Special Biological Resources in the Hanjiang River Basin, School of Life Sciences, Jianghan University, Wuhan 430056, China; lzy1377710@163.com (Z.L.);

**Keywords:** *Artemisia selengensis* Turcz. leaf, functional beer, fermentation dynamics, volatile compounds, metabolism

## Abstract

*Artemisia selengensis* Turcz. (AST), an edible-medicinal herb, contains multifunctional bioactives. This study investigated the application of AST leaf extract (ASTLE) in beer brewing, focusing on the addition stage and its impacts on fermentation dynamics, flavor profile, and functional properties. Fermentation parameters, bioactive compounds (phenolic; flavonoid), and volatiles (using HS-SPME-GC-MS) were analyzed. *In vivo* efficacy was assessed in high-fat diet-fed mice supplemented for 8 weeks with beer containing 10% ASTLE (post-primary fermentation), evaluating body weight change, serum lipids, and uric acid levels. It was found that adding ASTLE before primary fermentation promoted yeast activity but increased the risk of excessive diacetyl production. Adding ASTLE after primary fermentation significantly increased total phenolic and flavonoid contents. GC-MS analysis revealed that ASTLE contributed 28 additional volatile compounds, including chrysanthenone and eucalyptol, thereby enriching the beer’s flavor profile and complexity. In mice, beer with 10% ASTLE (post-primary fermentation) reduced body-weight gain, and regulated abnormal blood lipids and serum uric acid levels. Adding ASTLE after primary fermentation optimized fermentation stability, bioactive retention, flavor enhancement, and conferred benefits including body-weight regulation, lipid metabolism improvement, and uric acid control, providing a reference for developing functional beers targeting health-conscious consumers.

## 1. Introduction

Gout is a disease caused by the long-term elevation of uric acid (UA) levels in the blood, leading to the crystallization and deposition of urate in joints and other tissues. Thus, hyperuricemia is recognized as the most critical biochemical basis and direct pathogenic factor for gout attacks [1,2]. Existing studies indicate that plasma purine concentrations increase significantly after beer consumption, providing a physiological foundation for hyperuricemia and gout [3]. Additionally, beer itself is high in calories, long-term excessive intake may lead to energy surplus, obesity, dyslipidemia, and increased cardiovascular risk [4]. As a long-established fermented beverage, beer boasts a vast consumer base due to its appealing flavor and social attributes, while also representing an integral component of dietary culture. Today, amid the growing conflict between consumers’ increasing demand for healthy diets and the potential health risks posed by traditional beer, more breweries are focusing on producing low-calorie or low-alcohol beers [5]. Developing functional beer products that offer both health benefits and desirable flavors will be a crucial research direction.

Historically, plants from the Artemisia genus, such as *A. absinthium* (wormwood) and *A. vulgaris* (mugwort), were fundamental components of traditional “gruit” in medieval Europe, prized for their bittering, aromatic and preservative properties prior to the dominance of hops [6]. However, the exploration of such “botanical additives” in the context of modern functional brewing remains limited, warranting in-depth investigation into their health benefits and complex flavor profiles.

*Artemisia selengensis* Turcz. (AST), a perennial herb of the *Artemisia* genus in the Asteraceae family, is widely distributed in the middle and lower reaches of the Yangtze River in China. It possesses not only a unique, intense aroma but also significant nutritional and medicinal value [7,8]. Unlike its historical relatives like mugwort or the regulated wormwood, AST is primarily valued as a seasonal edible vegetable. Its tender stems are highly appreciated for their unique flavor and texture in local cuisine. It is noteworthy that while its stems are commonly consumed, other parts are often discarded, resulting in low utilization rates. Recent studies highlight that AST leaf are rich in high-value bioactive compounds, including flavonoids, polyphenols, and terpenoids [9,10]. *In vitro* and animal experiments confirm that *Artemisia selengensis* leaf extract (ASTLE) exhibits notable antioxidant, anti-inflammatory, and xanthine oxidase (XOD)-inhibiting effects [11,12,13]. Moreover, ASTLE alleviates dyslipidemia in mice induced by a high-fat diet [14]. Therefore, utilizing these often-discarded resources to develop functional foods or additives holds substantial economic and health significance. Incorporating ASTLE into beer may not only impart distinctive flavors but also confer potential health-functional value, offering a novel approach to mitigate the health concerns associated with traditional beer.

However, beer fermentation is a complex process. The impact of adjunct additions on yeast growth and metabolism remains unclear [15]. Furthermore, it is essential to verify whether bioactive components in ASTLE such as polyphenols and flavonoids remain stable during fermentation and whether their health benefits persist in the final product. Currently, no studies have reported the application of AST or its extracts in beer brewing.

To explore the feasibility of utilizing AST in brewing and preliminarily evaluate its health value, this study investigates the effects of ASTLE addition on beer fermentation dynamics (yeast proliferation, sugar metabolism, ethanol production, diacetyl evolution); the influence of addition timing on the bioactive compound content (total phenolics, total flavonoids) of the final product; the volatile flavor profile of AST beer and the regulatory efficacy of AST beer on body weight, serum lipids, and UA levels in high-fat diet-fed mice. This work aims to provide foundational support for developing related functional products.

## 2. Results and Discussion

### 2.1. Effects of ASTLE on Primary Fermentation

**Figure 1:**
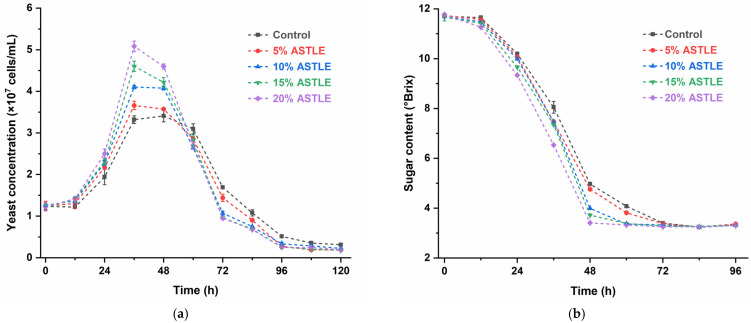

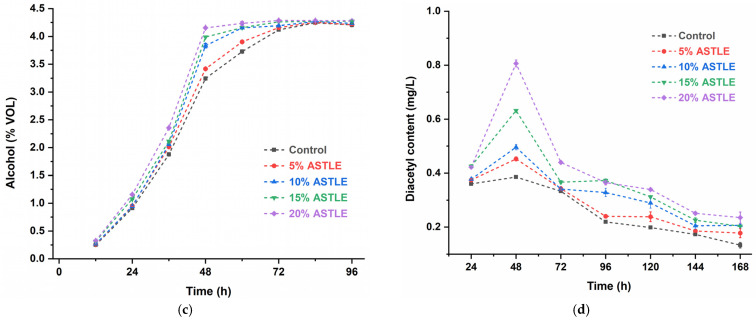
Effect of ASTLE on primary fermentation. (**a**) Yeast concentration; (**b**) Sugar content; (**c**) Alcohol content; (**d**) Diacetyl content. Data are expressed as the means ± SE (n = 3).

Adding ASTLE before primary fermentation significantly influenced beer fermentation. As shown in Figure 1a, compared to the control group (0% ASTLE), experimental groups with ASTLE exhibited enhanced yeast growth rates and higher maximum cell concentrations, with effects intensifying proportionally to ASTLE dosage. At 36 h, the 20% ASTLE group reached its peak yeast concentration (5.083 ± 0.123 × 10^7^ cells/mL), while the control group peaked later at 48 h (3.407 ± 0.142 × 10^7^ cells/mL).

Sugars in the fermentation system are primarily utilized by yeast for proliferation, metabolic maintenance, and ethanol synthesis. Figure 1b,c demonstrate that accelerated yeast proliferation in ASTLE-supplemented groups led to faster fermentable sugar consumption and earlier ethanol peak attainment. These results indicate that adding ASTLE before primary fermentation enhances yeast growth and accelerates early-stage fermentation in a concentration-dependent manner. This result aligns with established findings that phenolic and flavonoid compounds derived from grape seeds enhance yeast fermentative activity [16].

Diacetyl content is a key indicator of beer maturity [17]. While appropriate diacetyl levels (≤0.15 mg/L) contribute to flavor complexity, excess amounts impart a sour or rotten off-flavor [18]. Diacetyl precursors (α-acetolactate) are byproducts of valine biosynthesis. During early fermentation, diacetyl accumulates with rapid yeast growth; later, yeast cells reabsorb and reduce diacetyl to less odorous 2,3-butanediol via dehydrogenases [19]. In this study (Figure 1d), adding ASTLE before primary fermentation elevated diacetyl levels at 48 h proportionally to dosage. The 20% ASTLE group peaked at (0.806 ± 0.012 mg/L), significantly higher than the control (0.386 ± 0.003 mg/L). This correlates with accelerated yeast proliferation (Figure 1a), which increased diacetyl production via enhanced valine biosynthesis. By the end of primary fermentation (168 h), only the control group reduced diacetyl below 0.15 mg/L (0.133 ± 0.010 mg/L). Other groups still retained elevated levels, with the 20% ASTLE group at (0.235 ± 0.021 mg/L). Notably, yeast concentration in this group (0.189 ± 0.002 × 10^7^ cells/mL) was significantly lower than the control group (0.310 ± 0.026 × 10^7^ cells/mL). Reduced yeast viability impeded diacetyl reduction, potentially extending production cycles or even causing reduction failure.

In summary, adding ASTLE before primary fermentation accelerates early-stage fermentation, offering pathways to improve brewing efficiency or reduce initial yeast pitching. However, the risk of excessive diacetyl production requires careful consideration. Furthermore, the stability of bioactive compounds in the finished beer must be evaluated.

### 2.2. Effects of ASTLE Addition Timing on Total Phenolic and Flavonoid Contents

**Figure 2:**
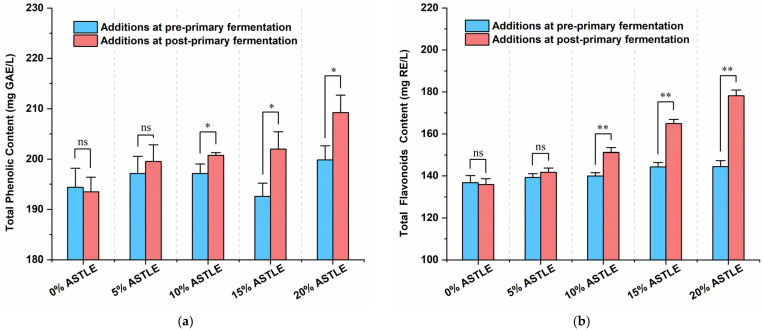
Total phenolic and flavonoid content of beer samples, added ASTLE at different fermentation stage. (**a**) Total phenolic content; (**b**) Total flavonoid content. Data are expressed as the means ± SE (n = 3). Asterisks indicate significant differences between pre- and post-fermentation addition at each dosage, as determined by Student’s *t*-test. * *p* < 0.05; ** *p* < 0.01; ns, not significant.

The content of bioactive compounds, particularly phenolics and flavonoids, is a key parameter in functional beer development, as these compounds are strongly correlated with beneficial properties [20,21]. As shown in Figure 2a,b, ASTLE added post-primary fermentation resulted in significantly higher total phenolic content (*p* < 0.05) and markedly elevated total flavonoids (*p* < 0.01) compared to pre-primary fermentation addition. Both parameters also increased with increasing ASTLE dosage.

Literature suggests that flavonoids can be enzymatically converted to phenolic acids during fermentation [22]. Similarly, phenolics and flavonoids derived from ASTLE may be depleted by yeast metabolism. Moreover, due to their instability [23], these active components may adsorb onto cell surfaces and co-sediment with the cells [16]. Consequently, pre-primary fermentation addition resulted in a lower final content of these bioactive compounds. In contrast, post-primary fermentation addition avoids this loss, leading to a dose-dependent increase in both total phenolics and flavonoids. Critically, as established earlier, post-primary fermentation addition also prevents adverse effects on fermentation dynamics (such as excessive diacetyl production) associated with pre-primary addition. Therefore, by mitigating fermentation risks while maximizing the content of bioactive compounds, post-primary fermentation addition represents a more effective process design.

### 2.3. Volatile Compound Analysis of AST Beer

Volatile flavor compounds are critical determinants of beer sensory quality. To assess the impact of ASTLE on beer flavor, HS-SPME-GC-MS was employed to analyze control beer (0% ASTLE) and AST beer (10% (*w*/*w*) ASTLE, post-primary fermentation), both prepared according to the described methodology. Results are summarized in Table 1.

In the control beer, 73 volatile compounds were identified. The profile was dominated by alcohols (total relative content 56.60%), and esters (33.68%), followed by acids (6.42%), alkanes (2.95%), and aldehydes (0.12%). In contrast, AST beer exhibited significantly enhanced volatile complexity with 91 compounds detected. While alcohols (52.31%), esters (33.62%), and acids (9.86%) remained major classes, ketones (2.54%) and olefins (0.05%)—undetected in controls—emerged as novel components. Concurrently, alkanes (1.15%) and aldehydes (0.19%) contributed minimally. This restructuring of the volatile profile confirms that ASTLE addition diversifies aromatic compounds and enriches sensory complexity.

Comparative analysis identified 10 compounds present in the control beer but undetected in the AST beer, including: 2-ethyl-1-hexanol, 3-furanmethanol, citronellol, n-heptadecanol-1, decanal, 2-methyl-propanoic acid, butanoic acid, acetic acid octyl ester, (Z)-3,7-dimethyl-2,6-octadien-1-ol acetate, and heptadecanoic acid ethyl ester. Notably, decanal, a common aldehyde in food flavors associated with waxy or tallow off-notes and primarily derived from lipid oxidation [24], was absent in the AST beer. This absence may be attributable to the enhanced antioxidant activity conferred by ASTLE. Conversely, 28 novel compounds not detected in the control beer were identified in the AST beer. Most of which were described as having flavor characteristics by herb, floral, woody, and mint notes. Among the added compounds, chrysanthenone (1.76%) and eucalyptol (1.21%) were dominant; both are widely reported flavor constituents in Asteraceae plants [25,26]. Chrysanthenone is commonly associated with Asteraceae-type aroma notes, while eucalyptol has been described as contributing cooling sensations. Although the actual sensory perception in beer remains to be confirmed by dedicated sensory evaluation, these unique volatiles indicate that ASTLE addition introduced characteristic compounds not present in the control beer, thereby enriching the overall flavor composition and complexity of the product.

### 2.4. Effects of AST Beer on High-Fat Diet-Fed Mice

To evaluate the long-term regulatory effects of AST beer on serum lipids and UA levels, a hyperlipidemic mice model was established by 8-week high-fat diet (HFD) feeding. Daily gavage interventions were administered, with body weight changes recorded throughout the experiment. At termination, serum levels of T-CHO, TG, LDL-C, HDL-C, and UA were measured.

#### 2.4.1. Effects on Body Weight and Serum Lipid Levels

**Figure 3:**
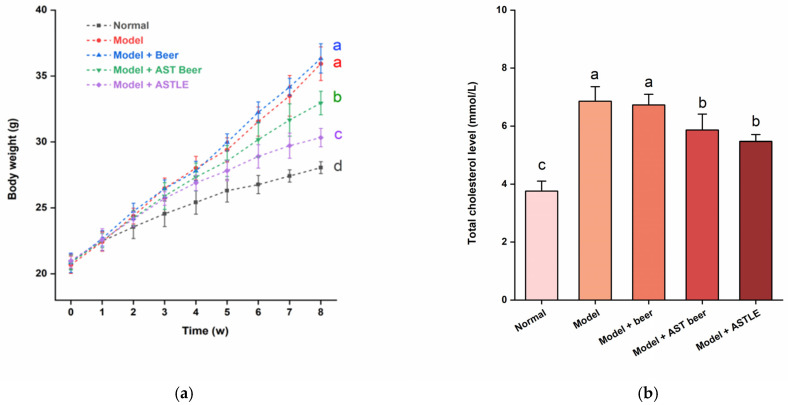

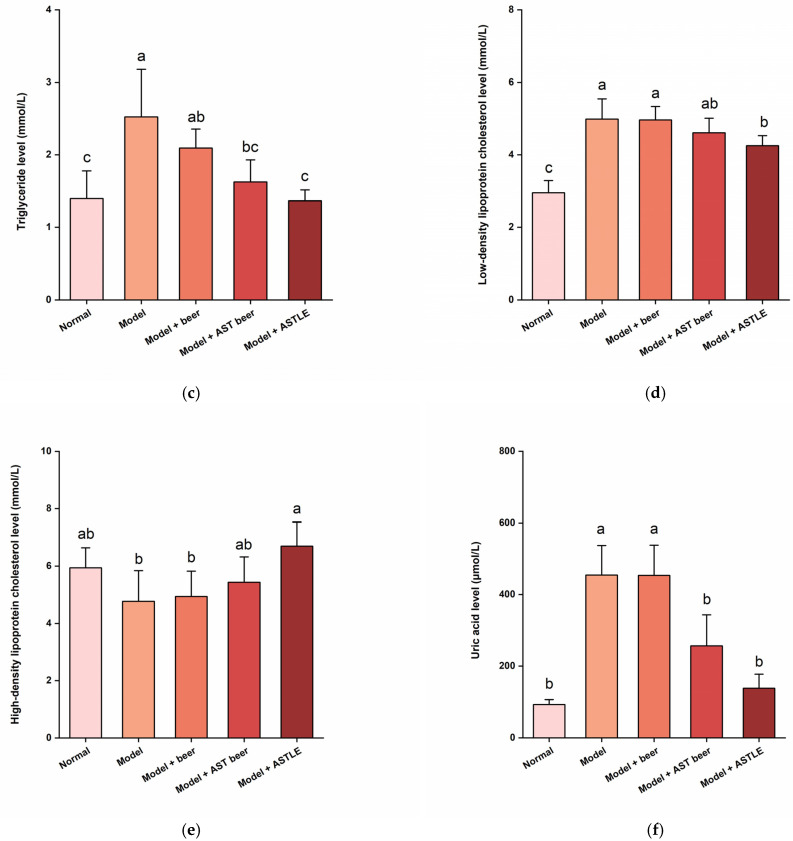
Effects of AST beer and other samples on physiological parameters in high-fat diet-fed mice. (**a**) Body weight gain trajectory over 8-week intervention; (**b**) Total cholesterol (TC) levels; (**c**) Triglyceride (TG) levels; (**d**) Low-density lipoprotein cholesterol (LDL-C) levels; (**e**) High-density lipoprotein cholesterol (HDL-C) levels; (**f**) Uric acid (UA) levels. Data are expressed as the means ± SE (n = 6). Different letters represent statistical significance (*p* < 0.05), as determined by ANOVA.

As shown in Figure 3a, all groups exhibited body weight gain during the study period. Compared with the Normal group fed with standard maintenance feed, HFD-fed groups showed significantly higher weight gain rates (*p* < 0.05). Mice administered beer containing 0% ASTLE by oral gavage (Model + beer group) showed higher average weight gain than the Model group, although this difference was not statistically significant (*p* > 0.05). In contrast, direct oral gavage of ASTLE (Model + ASTLE group) significantly reduced weight gain by 15.58% compared to the Model group (*p* < 0.05). While the intervention effect of AST beer (Model + AST beer group) was weaker than that of direct ASTLE gavage, it still resulted in a significant 8.30% reduction in average weight gain compared to the Model group (*p* < 0.05), effectively mitigating HFD-induced weight gain.

Weight gain induced by HFD is commonly accompanied by lipid dysregulation. As shown in Figure 3b–e, the Model group exhibited pronounced dyslipidemia when compared to the Normal group. The levels of TC, TG, and LDL-C significantly increased by 82.40%, 80.75%, and 68.80% (*p* < 0.05), alongside a 19.72% reduction in HDL-C (*p* < 0.05). These results indicate that HFD successfully induced dyslipidemia in mice. Administration of beer without ASTLE (Model + beer group) via oral gavage showed no statistically significant alterations in TC, TG, LDL-C, or HDL-C levels relative to the Model group (*p* > 0.05), indicating that the base beer formulation lacks efficacy against HFD-induced dyslipidemia. On the contrary, direct oral gavage of ASTLE (Model + ASTLE group) significantly ameliorated all lipid parameters versus the Model group (*p* < 0.05). Reductions of 20.21% in TC, 45.83% in TG, and 14.68% in LDL-C were observed, while HDL-C increased by 40.35%. These results demonstrate potent lipid-regulating properties of ASTLE. AST beer intervention (Model + AST Beer group) similarly improved lipid profiles relative to the Model group, yielding reductions of 14.41% in TC, 35.49% in TG, and 7.60% in LDL-C, with a 14.04% HDL-C elevation. Statistical significance (*p* < 0.05) was achieved for TC and TG reductions, though LDL-C and HDL-C changes did not reach significance (*p* > 0.05). Although AST beer’s efficacy was less pronounced than ASTLE, it still retained significant lipid-regulating functions.

The mitigation of HFD-induced weight gain and dyslipidemia by ASTLE and AST beer suggests an improvement in overall metabolic health. Recent studies have extensively documented the lipid-regulating properties of Artemisia genus. The di-caffeoylquinic acid (di-CQA) and its derivatives abundant in AST are demonstrated efficacy in enhancing intracellular fatty acid oxidation [27]. Concurrently, polyphenolic compounds modulate gut microbiota composition by promoting beneficial bacterial populations, thereby mitigating obesity-related dyslipidemia [28,29]. Further evidence indicates that AST extracts suppress triglyceride and cholesterol synthesis through downregulation of key biosynthetic genes [14]. Based on the lipid-modulating mechanisms described above, our study incorporated ASTLE at the end of primary fermentation to confer lipid-regulating functionality to beer. Experimental results confirm that despite potential interference from beer, the lipid-modulating efficacy of ASTLE was preserved in the finished beer. Furthermore, these results indicate that AST beer supplementation contributed to improved metabolic outcomes, including the moderation of weight gain.

#### 2.4.2. Effects of AST Beer on Serum UA Levels

β-Oxidation of fatty acids upregulates UA synthesis-related genes, promoting UA production [30]. HFD amplifies this process, leading to dyslipidemia and elevated serum uric acid levels. As shown in Figure 3f, UA in the Model group was significantly elevated by 390.36% compared to the Normal group (*p* < 0.05), confirming HFD induced dysregulation of UA homeostasis in mice. In the AST beer treatment group, serum UA levels were decreased significantly by 43.49% compared to the Model group (*p* < 0.05), and the ASTLE group showed more pronounced reductions of 69.53% relative to the Model group (*p* < 0.05).

Recent studies reveal that ethanol extracts of AST leaf significantly reduce serum UA levels in mice without inducing hepatorenal toxicity [31]. This effect is potentially mediated by 1,4-diCQA and related compounds abundant in AST leaf, which compete for binding at the enzymatic active site of XOD, the key enzyme in UA biosynthesis, this molecular interaction induces conformational changes in XOD’s secondary structure, thereby suppressing UA production [32].

It is noteworthy that compared with the model group, the consumption of control beer (0% ASTLE) did not significantly increase serum UA levels. This finding appears contradictory to the well-documented potential of beer to elevate serum UA levels in epidemiological studies. Typically, beer induced serum UA levels elevation is attributed to its purine content and ethanol, with this effect being most pronounced within a specific time window after ingestion [3]. In the present study, however, blood sampling was performed after a 10 h fasting period (aiming to minimize dietary interference), by which time serum UA fluctuations triggered by beer consumption may have been partially metabolized. Furthermore, the chronically elevated serum UA levels induced by long-term HFD in this model might have masked the effects of the administered beer dose. 

Importantly, this context makes the significant reduction in UA by AST beer even more compelling. A key challenge in developing AST beer for hyperuricaemic populations is to balance two opposing factors: the UA-regulating capacity conferred by ASTLE and the inherent hyperuricaemic risk posed by beer constituents such as ethanol and purines. In our study, at a dose where the control beer (with an equivalent ethanol content) showed no significant effect, the AST beer containing 10% (*w*/*w*) ASTLE significantly lowered serum UA levels in HFD-fed mice. This directly demonstrates that AST beer retains the urate-lowering property of ASTLE. Nevertheless, the efficacy of this effect in humans and the optimal dosage will require confirmation in clinical trials.

## 3. Materials and Methods

### 3.1. Materials

#### 3.1.1. Raw Materials

Fresh AST leaves were harvested from the Caidian Base of Jianghan University (Wuhan, China). Barley malt (Pale Ale Malt) was purchased from Malteurop (Baoding) Malting Co., Ltd. (Baoding, China). Hops (Cascade) were obtained from Yakima Valley (Washington, DC, USA). Saccharomyces cerevisiae yeasts (S-33) were sourced from Fermentis (Lesaffre Group, Ghent, Belgium). Brewing water was standard municipal tap water supplied by Jianghan University (Wuhan, China).

#### 3.1.2. Reagents and Standards

Folin–Ciocalteu reagent were purchased from Phygene Biotechnology Co., Ltd. (Fuzhou, China). Rutin was purchased from Shanghai Yuanye Biotechnology Co., Ltd. (Shanghai, China). Gallic acid was acquired from Shanghai Macklin Biochemical Co., Ltd. (Shanghai, China). Sodium carbonate, sodium nitrite, aluminum nitrate nonahydrate, sodium hydroxide, and o-phenylenediamine were purchased from Sinopharm Chemical Reagent Co., Ltd. (Beijing, China). Reagent water used was ultrapure deionized water. Assay kits for the determination of UA, total cholesterol (T-CHO), triglycerides (TG), low-density lipoprotein cholesterol (LDL-C), and high-density lipoprotein cholesterol (HDL-C) were obtained from Nanjing Jiancheng Bioengineering Institute (Nanjing, China).

### 3.2. Brewing Process

#### 3.2.1. Preparation of ASTLE

Fresh AST leaves were washed, surface-dried, and mixed with 10-fold (*w*/*w*) deionized water. The mixture was homogenized for cell disruption with a high-speed blender (ANS-1680B, Kamedror, Guangzhou, China). The homogenate was filtered through a 200-mesh sieve, and the resulting extract was sealed and stored at 4 °C for later use.

#### 3.2.2. Preparation of Wort

In this study, all wort was prepared as follows (equipment pre-sterilized): First, Barley malt was lightly moistened and crushed using a two-roller mill. Secondly, crushed malt was mixed with water at 45 °C (malt-to-water ratio 1:3, *w*/*w*) in a mash tun and stirred for 10 min. Next, the temperature was increased to 68 °C at a rate of 1 °C/min and then held for 40 min, followed by heating to 78 °C at 1 °C/min to terminate saccharification. The mash was transferred to a lauter tun for solid–liquid separation. First wort was collected, and residual solids were sparged with 78 °C water. Combined wort was adjusted to 14.5 ± 0.5°Bx using a handheld refractometer. After lautering, the wort was boiled (100 °C) for 65 min and Cascade hops were added at 20 min and 40 min after the start of boiling, yielding a total hop addition of 0.10% (*w*/*w* of total wort). After boiling, stir the wort to make it swirl and then standing for 20 min. The supernatant was transferred to fermentation bottles (2.8 kg/bottle) and cooled to 20 °C in an incubator.

#### 3.2.3. ASTLE Addition and Fermentation

Each group consists of 5 bottles of wort, and 0 g, 175 g, 350 g, 525 g, or 700 g of ASTLE were added and then used deionized water to adjust total mass to 3.5 kg/bottle (equivalent to 0%, 5%, 10%, 15%, 20% *w*/*w*, respectively). Take another group of 5 bottles of wort and identical ASTLE doses added after primary fermentation, with mass adjusted to 3.5 kg/bottle. The yeast (S-33) was inoculated at 0.25 g/kg wort. Primary fermentation proceeded at 20 °C for 7 days, followed by cold storage at 4 °C for 7 days. Each pair of groups constituted one fermentation batch, and the fermentation experiments were replicated three times.

### 3.3. Fermentation Parameter Monitoring

The yeast concentration was measured every 12 h using an automated cell counter (RWD-C100, RWD Life Science Co., Shenzhen, China). Residual sugar and ethanol content were analyzed every 12 h using FermentoFlash (Funke Gerber, Berlin, Germany). The level of diacetyl was quantified daily according to Chinese National Standard GB/T 4928-2008 [33].

### 3.4. Analysis of Bioactive Compounds

#### 3.4.1. Determination of Total Polyphenol Content

Total polyphenol content (TPC) in beer was determined using the Folin–Ciocalteu spectrophotometric method as described previously [34] with minor modifications. Briefly, 0.1 mL of degassed beer sample was mixed with 1 mL of Folin–Ciocalteu reagent. After 5 min of the reaction, 1 mL of 7.5% Na_2_CO_3_ aqueous solution and 2.75 mL deionized water were added. The mixture was incubated at room temperature for 60 min, vortexed to remove bubbles, and absorbance measured at 765 nm using an ultraviolet spectrophotometer (UV-5100B, Shanghai Metash Instruments Co., Ltd., Shanghai, China). TPC was calculated from a gallic acid standard curve (50–500 mg/L) and expressed as gallic acid equivalents (GAE, mg/L). All values represent the mean of triplicate measurements.

#### 3.4.2. Determination of Total Flavonoid Content

Total flavonoid content (TFC) was quantified using the aluminum nitrate colorimetric method [35] with modifications. Briefly, 5 mL degassed beer sample was mixed with 0.5 mL of 5% NaNO_2_ solution. After 6 min of the reaction, 0.5 mL of 10% Al(NO_3_)_3_ solution was added and mixed. The mixture was incubated at room temperature for 6 min, followed by addition of 4 mL of 4% NaOH solution. After 20 min for reaction, the absorbance was measured at 510 nm with an ultraviolet spectrophotometer. TFC was calculated from a rutin standard curve (40–200 mg/L) and expressed as rutin equivalents (RE, mg/L). All values represent the mean of triplicate measurements.

### 3.5. GC-MS Analysis

Control beer (0% ASTLE) and AST beer (10% (*w*/*w*) ASTLE, post-primary fermentation) samples were prepared according to the method outlined in Section 3.2.3. Volatile compounds were extracted by headspace solid-phase microextraction (HS-SPME) and analyzed via GC-MS (QP2020NX-GC2030, Shimadzu Corporation, Kyoto, Japan). A 10 mL aliquot of non-degassed beer was transferred to a 45 mL headspace vial with 2 g NaCl. The sample was incubated at 50 °C for 10 min, followed by 30 min extraction. Separation used a DB-5MS capillary column (60 m × 0.25 mm × 0.25 μm) with helium (99.999%) carrier gas at a constant flow rate of 1.0 mL/min. The injector temperature was 250 °C. The initial temperature was 40 °C, maintained for 3 min, then increased to 80 °C at a rate of 4 °C/min, maintained for 5 min, and finally ramp at 5 °C/min to 240 °C, hold 10 min. Mass spectrometry conditions: ion source temperature was 230 °C, electron ionization energy 70 eV, quadrupole 150 °C, transfer line 240 °C, scan range 30–450 amu. Compounds were identified by matching against the NIST20 mass spectral library, retaining results with similarity index (SI) >85. Relative content was analyzed by peak area normalization.

### 3.6. Animal Experiments

Male Balb/c mice (SPF, 19–22 g) were supplied by Wuhan Cloud-Clone Animal Technology Co., Ltd. (License No. SYXK(Hubei) 2023-0135; Wuhan, China). All procedures complied with the Cloud-Clone Ethics Committee regulations (Ethics No. IACU24-1402). Mice were housed under a controlled temperature of 23 ± 3 °C and 55 ± 15% relative humidity with a 12 h light/dark cycle. The animals were acclimated to the environment for one week and were fed with pellet diets and purified water during the whole experimental period.

The mice were randomly divided into 5 groups (n = 6/group) on day 7: Normal group, Model group, Model + beer group, Model + AST beer group, Model + ASTLE group.

The Normal group was fed a standard maintenance diet, while all other groups received a high-fat diet (XT301-1, Jiangsu Xietong Pharmaceutical Bio-Engineering Co., Ltd., Nanjing, China). All groups received daily oral gavage at a dose of 10 mL/kg body weight. The Normal and Model groups received physiological saline; the Model + beer group received control beer (0% ASTLE); the Model + AST beer group was given AST beer (10% (*w*/*w*) ASTLE, post-primary fermentation); and the Model + ASTLE group received ASTLE. The beer samples and ASTLE were prepared as described in Section 3.2.3 and Section 3.2.1, respectively.

Body weight was recorded weekly. After 8 weeks, mice were fasted for 10 h, anesthetized with sodium pentobarbital, and subjected to orbital blood sampling followed by cervical dislocation. Serum was separated and analyzed for UA, T-CHO, TG, LDL-C, and HDL-C levels using commercial kits.

### 3.7. Statistical Analysis

Statistical analyses were performed using SPSS Statistics 26.0 (IBM Corp., Armonk, NY, USA). Data were expressed as mean ± standard deviation. Comparisons between two independent groups were conducted using Student’s *t*-test. For multiple group comparisons, one-way ANOVA was applied. Post hoc analyses used Tukey’s test (if variance was homogeneous) or Tamhane’s T2 test (if variance was heterogeneous). A *p*-value < 0.05 was considered statistically significant. Graphs were generated with OriginPro 2021 (OriginLab Corp., Northampton, MA, USA).

## 4. Conclusions

This study represents the first application of ASTLE to beer brewing, systematically evaluating its impact on fermentation dynamics, flavor profile, and functional properties. The principal findings are summarized as follows: (i) Addition of ASTLE before primary fermentation significantly influenced yeast metabolism. While it enhanced yeast proliferation, it concurrently elevated diacetyl levels and resulting in residual diacetyl exceeding organoleptic thresholds at fermentation endpoint. (ii) Introducing ASTLE at the end of primary fermentation effectively mitigated diacetyl-related risks while significantly increasing the content of bioactive compounds. (iii) ASTLE significantly enhances the beer’s aromatic complexity through the introduction of 28 distinct volatile compounds, notably chrysanthenone and eucalyptol, which collectively contribute to a more sophisticated flavor profile. (iv) The study also confirmed that beer containing 10% ASTLE (*w*/*w*, additions at post-primary fermentation) exhibits significant physiological benefits, including: body weight management, serum lipid regulation and UA control. In summary, incorporating ASTLE into brewing not only provides a method to improve the utilization of AST resources, but also supports the development of functional beer with unique flavors for health-conscious consumers.

## Figures and Tables

**Table 1:** Relative content of flavor compounds in different beers.

Category	Compounds	RI	CAS	Relative Content (%)	
				CK	AST Beer
Alcohols (32)	Ethanol	1968	64-17-5	35.01	31.17
	2-methyl-1-Propanol	2167	78-83-1	0.73	0.63
	3-methyl-1-Butanol	1136	123-51-3	10.08	8.71
	1-Heptanol	1869	111-70-6	0.06	0.06
	2-ethyl-1-Hexanol	1259	104-76-7	0.07	—
	2-Nonanol	1259	628-99-9	0.01	0.02
	3,7-dimethyl-1,6-Octadien-3-ol	1284	78-70-6	0.08	0.13
	1-Octanol	995	111-87-5	0.24	0.27
	3-Furanmethanol	785	4412-91-3	0.01	—
	1-Nonanol	1179	143-08-8	0.07	0.09
	3-(methylthio)-1-Propanol	1228	505-10-2	0.01	0.01
	2-Tridecanol	1710	1653-31-2	0.01	0.02
	1-Decanol	1083	112-30-1	0.24	0.26
	Citronellol	1183	106-22-9	0.05	—
	(Z)-3,7-dimethyl-2,6-Octadien-1-ol	1580	106-25-2	0.01	0.01
	10-Undecen-1-ol	1447	112-43-6	0.01	0.01
	Phenylethyl Alcohol	775	60-12-8	9.71	8.85
	1-Dodecanol	1035	112-53-8	0.06	0.08
	(E)-3,7,11-trimethyl-1,6,10-Dodecatrien-3-ol	1814	40716-66-3	0.03	0.05
	1-Hexadecanol	1381	36653-82-4	0.06	0.11
	trans-Farnesol	860	106-28-5	0.04	0.05
	n-Heptadecanol-1	960	1454-85-9	0.01	—
	Eucalyptol	1059	470-82-6	—	1.21
	1-Hexanol	1179	111-27-3	—	0.03
	1-Octen-3-ol	1083	3391-86-4	—	0.07
	(R)-4-methyl-1-(1-methylethyl)-3-Cyclohexen-1-ol	1183	20126-76-5	—	0.18
	(Z)-2-(3,3-dimethylcyclohexylidene)ethan-1-ol	1258	26532-23-0	—	0.10
	6-Octen-1-ol, 3,7-dimethyl-, (R)-	1204	1117-61-9	—	0.08
	Ledol	1347	577-27-5	—	0.05
	alpha-Cadinol	1457	481-34-5	—	0.01
	2-(dodecyloxy)-Ethanol	2175	4536-30-5	—	0.01
	(E)-(±)-3,7,11-trimethyl-6,10-Dodecadien-1-ol	1507	20576-54-9	—	0.04
Aldehydes (5)	Nonanal	1186	124-19-6	0.04	0.02
	Decanal	1913	112-31-2	0.02	—
	2,4-dimethyl-Benzaldehyde	1282	15764-16-6	0.06	0.06
	2,6,6-trimethyl-1,3-Cyclohexadiene-1-carboxaldehyde	697	116-26-7	—	0.06
	(±)-1,3,3-Trimethylcyclohex-1-ene-4-carboxaldehyde	984	127128-60-3	—	0.05
Ketones (4)	(-)-Carvone	1190	6485-40-1	—	0.21
	4,6,6-trimethyl-Bicyclo[3.1.1]hept-3-en-2-one	1137	80-57-9	—	0.23
	(S)-3-methyl-6-(1-methylethenyl)-2-Cyclohexen-1-one	1359	16750-82-6	—	0.34
	Chrysanthenone	1661	473-06-3	—	1.76
Acids (15)	Acetic acid	820	64-19-7	0.08	0.02
	2-methyl-Propanoic acid	1779	79-31-2	0.01	—
	Butanoic acid	1173	107-92-6	0.02	—
	3-methyl-Butanoic acid	1104	503-74-2	0.05	0.04
	Octanoic acid	1175	124-07-2	4.00	5.57
	n-Decanoic acid	2077	334-48-5	1.39	2.33
	9-Decenoic acid	1352	14436-32-9	0.24	0.37
	Dodecanoic acid	974	143-07-7	0.12	0.13
	Tetradecanoic acid	984	544-63-8	0.06	0.06
	Pentadecanoic acid	1570	1002-84-2	0.01	0.01
	n-Hexadecanoic acid	1159	57-10-3	0.17	0.19
	Octadecanoic acid	1362	57-11-4	0.15	0.15
	Oleic Acid	1954	112-80-1	0.12	0.14
	Hexanoic acid	1208	142-62-1	—	0.82
	9-Hexadecenoic acid	1476	2091-29-4	—	0.03
Esters (30)	Butanoic acid ethyl ester	1976	105-54-4	0.05	0.03
	3-methyl-1-Butanol acetate	1615	123-92-2	11.67	9.40
	Hexanoic acid ethyl ester	1270	123-66-0	3.34	2.75
	Acetic acid hexyl ester	1281	142-92-7	0.07	0.07
	Heptanoic acid ethyl ester	1680	106-30-9	0.04	0.03
	Octanoic acid ethyl ester	1372	106-32-1	6.40	6.44
	Acetic acid octyl ester	969	112-14-1	0.03	—
	Nonanoic acid ethyl ester	1854	123-29-5	0.03	0.03
	Decanoic acid ethyl ester	1564	110-38-3	3.23	5.06
	Ethyl 9-decenoate	1878	67233-91-4	1.92	2.23
	(Z)-3,7-dimethyl-2,6-Octadien-1-ol acetate	885	141-12-8	0.01	—
	Acetic acid 2-phenylethyl ester	1731	103-45-7	5.70	5.56
	Dodecanoic acid ethyl ester	1059	106-33-2	0.56	1.28
	Pentadecanoic acid 3-methylbutyl ester	1119	2306-91-4	0.04	0.04
	Benzenepropanoic acid ethyl ester	1580	2021-28-5	0.02	0.02
	Ethyl 9-hexadecenoate	1133	54546-22-4	0.14	0.14
	Ethyl tridecanoate	1036	28267-29-0	0.01	0.01
	Heptadecanoic acid ethyl ester	811	14010-23-2	0.04	—
	dihydro-5-pentyl-2(3H)-Furanone	912	104-61-0	0.02	0.02
	Isopropyl myristate	1240	110-27-0	0.01	0.02
	Tetradecanoic acid ethyl ester	1769	124-06-1	0.12	0.10
	Pentadecanoic acid ethyl ester	1986	41114-00-5	0.03	0.02
	Hexadecanoic acid ethyl ester	1131	628-97-7	0.09	0.09
	1,2-Benzenedicarboxylic acid bis(2-methylpropyl) ester	1654	84-69-5	0.01	0.03
	Dibutyl phthalate	1860	84-74-2	0.10	0.16
	Acetic acid heptyl ester	1530	112-06-1	—	0.04
	5-methyl-2-(1-methylethenyl)-4-Hexen-1-ol acetate	1978	25905-14-0	—	0.01
	Benzeneacetic acid ethyl ester	1078	101-97-3	—	0.01
	2-methyl-Propanoic acid 3-hydroxy-2,4,4-trimethylpentyl ester	463	74367-34-3	—	0.02
	2-Ethylhexyl salicylate	576	118-60-5	—	0.01
Alkanes (5)	Dodecamethyl-cyclohexasiloxane	1190	540-97-6	1.55	0.01
	Tetradecamethyl-cycloheptasiloxane	1371	107-50-6	0.89	0.07
	Hexadecamethyl-cyclooctasiloxane	1579	556-68-3	0.47	0.52
	Octadecamethyl-cyclononasiloxane	1331	556-71-8	0.04	0.51
	Caryophyllene oxide	1082	1139-30-6	—	0.04
Olefins (2)	Verbenyl ethyl ether	597	823204-45-1	—	0.04
	Humulene	711	6753-98-6	—	0.01
Other (8)	4-Hydroxy-2-methylacetophenone	1119	875-59-2	0.06	0.06
	2,4-bis(1,1-dimethylethyl)-Phenol	1184	96-76-4	0.05	0.05
	2,3-dihydro-Benzofuran	1908	496-16-2	0.01	0.01
	1,2,3,4-tetramethyl-Benzene	2037	488-23-3	—	0.02
	[1S-(1.alpha.,3.alpha.,5.alpha.)]-6,6-dimethyl-2-methylene-Bicyclo[3.1.1]heptan-3-ol	1363	547-61-5	—	0.03
	Naphthalene	1345	91-20-3	0.05	0.06
	1-methyl-Naphthalene	1231	90-12-0	0.02	0.02
	1-(1H-pyrrol-2-yl)ethenone	1555	1072-83-9	0.01	0.02

Relative contents determined by peak area normalization. CK: control beer (0% ASTLE); AST beer: 10% (*w*/*w*) ASTLE, added post-primary fermentation. These beer samples were prepared as described in Section 3.2.3.

## Data Availability

The original contributions presented in this study are included in the article. Further inquiries can be directed to the corresponding author.
